# Urinary 1-Hydroxypyrene as a Biomarker of PAH Exposure in 3-Year-Old Ukrainian Children

**DOI:** 10.1289/ehp.7898

**Published:** 2005-10-20

**Authors:** Amy Pelka Mucha, Daniel Hryhorczuk, Andrij Serdyuk, Joseph Nakonechny, Alexander Zvinchuk, Serap Erdal, Motria Caudill, Peter Scheff, Elena Lukyanova, Zoreslava Shkiryak-Nyzhnyk, Natalia Chislovska

**Affiliations:** 1 Great Lakes Center for Occupational and Environmental Safety and Health and; 2 Division of Environmental and Occupational Health Sciences, University of Illinois at Chicago, School of Public Health, Chicago, Illinois, USA; 3 Institute of Hygiene and Medical Ecology, Kiev, Ukraine; 4 Institute of Medico-Ecological Problems, Chernivtsi, Ukraine; 5 University of Illinois at Chicago Louise Hamilton Data Management Center, Kiev, Ukraine; 6 Institute of Pediatrics, Obstetrics and Gynecology, Kiev, Ukraine

**Keywords:** air pollution, biomarker, children, environment, polycyclic aromatic hydrocarbons

## Abstract

Urinary 1-hydroxypyrene (1-OHP) is a biomarker of polycyclic aromatic hydrocarbon (PAH) exposure. We measured urinary 1-OHP in 48 children 3 years of age in Mariupol, Ukraine, who lived near a steel mill and coking facility and compared these with 1-OHP concentrations measured in 42 children of the same age living in the capital city of Kiev, Ukraine. Children living in Mariupol had significantly higher urinary 1-OHP and creatinine-adjusted urinary 1-OHP than did children living in Kiev (adjusted: 0.69 vs. 0.34 μmol/mol creatinine, *p* < 0.001; unadjusted: 0.42 vs. 0.30 ng/mL, *p* = 0.002). Combined, children in both cities exposed to environmental tobacco smoke in their homes had higher 1-OHP than did children not exposed (0.61 vs. 0.42 μmol/mol creatinine; *p* = 0.04; *p* = 0.07 after adjusting for city). In addition, no significant differences were seen with sex of the children. Our sample of children in Mariupol has the highest reported mean urinary 1-OHP concentrations in children studied to date, most likely due to their proximity to a large industrial point source of PAHs.

Polycyclic aromatic hydrocarbons (PAHs) are a group of three- and four-ring compounds that are formed as a result of incomplete combustion. Sources of environmental contamination can be both industrial and nonindustrial, with the most common sources being cigarette smoke, coal-fired utilities, steel plants, vehicle exhaust, wood-burning ovens and fireplaces, and charcoal-grilled and smoked food. The greatest exposures to PAHs generally occur via cigarette smoke, emissions from wood-burning ovens and fireplaces, and consumption of grilled and broiled foods [International Agency for Research on Cancer (IARC) 1987]. PAHs are ubiquitous in the environment and are found in many environmental media, including indoor and ambient air, soil, and diet. Children tend to have higher PAH exposure to air, soil, and dust than do adults because of child-specific behavior patterns, such as hand-to-mouth activity and more time spent close to and on the ground, as well as having a higher inhalation rate on a per unit body-weight basis compared with adults (Committee on Environmental Health 1999; Landrigan et al. 1998). PAHs have been shown to bind to the aryl hydrocarbon receptor and affect multiple systems (Nebert and Atlas 1978). In addition, they act as carcinogens in numerous animal species and are used as positive controls in skin painting cancer studies. PAHs have also been shown to be human carcinogens in occupational settings and have been found to be causally associated with skin and lung cancer. Coke oven emissions are classified as known human carcinogens [IARC 1987; U.S. Environmental Protection Agency (EPA) 2003a], of which PAHs are a major constituent. PAHs have also been shown in animals to cause humoral and cellular immune toxicity (Davila et al. 1996).

Because PAH exposure occurs as a mixture of compounds, and because pyrene is almost always found in this mixture, pyrene and its metabolite 1-hydroxypyrene (1-OHP) are considered appropriate surrogate markers of total PAH exposure (Jacob and Seidel 2002). Among the many PAH compounds, pyrene is emitted in large amounts and almost always found in the presence of other PAHs, acting as a surrogate marker for all PAHs (Viau 2002). In the body, pyrene is primarily metabolized via the cytochrome P450 1A1 (CYP1A1) enzymes and excreted in the urine as 1-OHP (Jacob and Seidel 2002). Therefore, 1-OHP can be assessed relatively easily in urine samples (Jongeneelen 1994). 1-OHP has been found to be a good short-term measure of exposure to PAHs. The half-lives of 1-OHP reported in the literature include between 6 and 35 hr (Jongeneelen et al. 1990), from 16 to 20 hr (Buchet et al. 1992), and more recently, 9.8 hr using volunteers and facial masks (Brzeznicki et al. 1997). In general, urinary 1-OHP represents the last 24 hr of cumulative PAH exposure (Jongeneelen 1994).

1-OHP has been validated as a biomarker of occupational exposure to PAHs, including in coke oven workers (Jacob and Seidel 2002; Jongeneelen 2001; Viau 2002). Concentrations have ranged from 0.3 to 25 μmol/mol creatinine in coke oven workers (Jongeneelen 2001; Levin 1995). 1-OHP levels, after environmental exposures, are lower by one to two orders of magnitude, depending on background exposures and smoking habits (Jongeneelen 2001).

Studies of childhood PAH exposure and measurement of urinary 1-OHP have almost entirely been with lower-level PAH contamination than in the present study. Overall, data are much more scarce for children’s environmental PAH exposure than for workers’ exposure. Studies in children have been done in several countries, with most identifying diet as the most important contributor to 1-OHP (Chuang et al. 1999; van Wijnen et al. 1996; Vyskocil et al. 2000) and other studies finding a role for traffic and environmental tobacco smoke (ETS) (Kanoh et al. 1993; Siwinska et al. 1998). A review of 1-OHP concentrations in children is presented in Table 1 and displayed in Figure 1.

Van Wijnen et al. (1996) determined the urinary 1-OHP levels in Dutch children living in five distinct areas with differing levels of PAHs in soil and ambient air, from background traffic releases to areas with mine tailings. The researchers investigated the influence of recent consumption of food with high PAH content, indoor and outdoor sources of PAHs, hand-to-mouth behavior, and play habits of children obtained through a questionnaire on urinary 1-OHP levels. Only indoor sources of PAHs showed a small, positive association with 1-OHP levels. The authors concluded that the possible ambient environment-related differences were potentially too small to be detected in the variations of the intake of PAHs from daily diet. In Japan, Kanoh et al. (1993) assessed urinary 1-OHP levels of school children in two areas of Tokyo along arterial roads and in one suburban area. Children living in the higher traffic areas had significantly higher 1-OHP levels than did the children in the less polluted area, by a factor of 1.1–1.6 (Kanoh et al. 1993). Zhao et al. (1992) assessed biologic exposure of a small group of school children in Beijing, where ambient air has significant PAH pollution, and 1-OHP levels were elevated, as shown in Table 1. Siwinska et al. (1998) found that 1-OHP levels in children in Poland increased because of exposure to ETS only in the case of mother’s smoking, but the differences were not significant (*p* > 0.05). Studies in North Carolina children found no statistically significant relationship between urinary PAH metabolites and the estimated daily doses derived from PAH concentrations in the relevant environmental media because of the great variability among individuals (Chuang et al. 1999). In another study of two small groups of children recruited from a kindergarten in a high-traffic-density area and from a kindergarten in a less contaminated area in Montreal, Canada (Vyskocil et al. 2000), no relationship was found between absorbed pyrene doses, by ingestion or by inhalation, and 1-OHP levels in urine. This was attributed to uncertainties in the estimates of PAH uptake from food and/or small sample size limiting statistical power of the study. A study conducted in a Czech city with four groups of children recruited from polluted (high traffic density) and non-polluted areas found seasonal variation in 1-OHP and attributed variation to differences in tobacco smoke exposure (Fiala et al. 2001).

In this study we investigated young children living in close proximity to a significant environmental point source of PAHs—a steel mill and associated coke oven; a map of Mariupol with PAH sources and where the study participants live is provided in Figure 2. This analysis thus offered the opportunity to investigate whether a large environmental exposure relates to a significant increase in the PAH biomarker 1-OHP.

## Materials and Methods

### Study site description.

**Figure 2:**
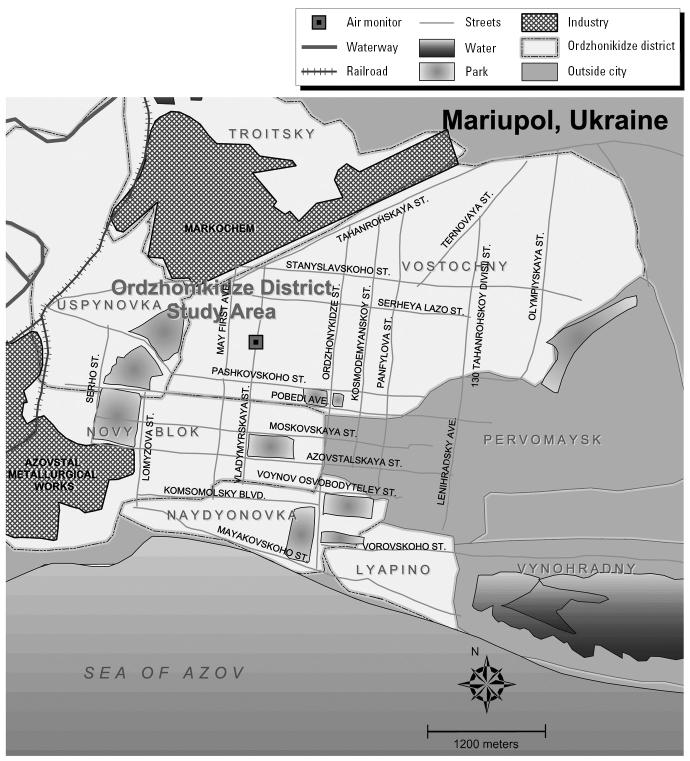
Map of Mariupol, Ukraine.

Mariupol is a city of approximately 530,000 in southeastern Ukraine, situated on the Azov Sea (Figure 2). Mariupol is considered one of the most heavily polluted cities in Ukraine, with multimedia contamination in the air, water, and soil. Mariupol’s landscape is dominated by two major steel plants and an associated coking facility. These plants use older equipment, some installed in the 1950s, with outdated technologies and minimal pollution control equipment. The two steel mills and coking facility combined are responsible for > 99% of air pollutants emitted from stationary sources in the city, two of which are located next to a residential area. The city’s coking facility is reported to emit > 30 kg of benzo[*a*]pyrene (BaP) annually into the atmosphere; the steel plants emit thousands of tons of nitrous oxides, carbon monoxide, and particulate matter [Ministry for Environmental Protection and Nuclear Safety of Ukraine (MEPNS) 1998]. Azovstal is the name of the steel mill and coke oven within 3 miles of the participants’ residences. A sample of children living in Kiev, the capital city of Ukraine with a population of 2.6 million, served as the comparison population.

### Epidemiologic design.

This study, Environmental Pollutants and Health Status of Children, was conducted as part of the Family and Children of Ukraine study, the Ukrainian component of the multicountry European Longitudinal Study of Pregnancy and Childhood (ELSPAC). ELSPAC is a prospective and geographically based series of population studies, which begin in pregnancy and follow the cohort of births until 7 years of age. The overall goal of the larger study is to identify risk factors for problems in pregnancy, reproductive outcomes, and childhood development (ELSPAC 1989).

In 1998, at the time of the ELSPAC assessment at 3 years of age, the parents of 884 children from Mariupol and 637 children from Kiev completed survey questionnaires. Children between 2.5 and 3.5 years of age were eligible for recruitment for participation in the Environmental Pollutants and Health Status of Children study, a cross-sectional morbidity study (*n* = 295 eligible; *n* = 244 enrolled: Mariupol, *n* = 171; Kiev, *n* = 73). Within this subset, 48 children from Mariupol and 42 children from Kiev were randomly selected for urinary measurement of 1-OHP. The biologic exposure assessment study coincided with the administration of the ELSPAC questionnaire for 3-year-old children; data from this questionnaire were included for analysis. In addition, a supplementary questionnaire on immune status was also implemented.

Children participating in this biomarker study received the ELSPAC questionnaire for 3-year-olds, abstracts of medical records for children 18 months to 3 years of age, and a supplementary immune health questionnaire. The ELSPAC 3-year-old questionnaire collected data on general health and medical treatment, diet, social and language development, and the child’s environment. This questionnaire was designed as a self-administered instrument to be completed by the mother or guardian. A trained district pediatrician from the local polyclinic then reviewed the questionnaire for completeness and inaccuracies and supplemented unanswered questions through an interview with the mother. The immune questionnaire was adopted from methods described by Straight et al. (1994). The immune questionnaire covers specific immune-related diseases and symptoms such as allergy symptoms, diagnosed infectious disease, and antibiotic use. This questionnaire was designed to be administered as an interview of the mother or guardian and was given to the parent bringing the child into the clinic for the assessment.

Data on age, sex, and second-hand smoke exposure were derived from these questionnaires. All questionnaires were translated and reverse translated for accuracy and delivered by native speakers to the children and parent(s). Questionnaire data could not be obtained from one study participant from Kiev.

### Collection and measurement of urinary 1-OHP.

Biomarker collection occurred during 16–21 March 1998 in Mariupol and 24–26 March 1998 in Kiev. A few days before field implementation of the study, urine sample collection receptacles were provided to the families by a nurse from the health clinics with instructions to collect first morning urine samples on the day of attending the clinic. The samples were kept at room temperature until delivered to the clinic. Nurses also answered questions and provided information on when the child and parent were to come to the clinic for further evaluation and sample delivery. Biologic samples were either received or collected on the same day as reporting to the clinic. No participant objected to providing urine samples, although six children did not provide enough urine for sample analysis (*n* = 90).

Collected urine samples were kept frozen at 18°C until transported on dry ice to the Institute of of Occupational Medicine and Environmental Health laboratory (Sosonowiecz, Poland) for sample analysis. The analysis method employed for 1-OHP detection is a reverse-phase high-performance liquid chromatography method with enzymatic hydrolysis, using β-glucuronidase/arylsulfatase (Jongeneelen et al. 1987). Creatinine, a clearance protein that adjusts for differences in urinary concentration, was also measured at the same laboratory.

### Ambient air analysis.

Ambient air sampling was conducted at one site in Mariupol, in an area determined to be representative of study participants’ exposure, based on wind variability data. Samples were collected using 37-mm quartz filters and measured particulate-phase PAHs only. The following PAHs were measured in the 10 μm fraction of particulate matter (PM_10_): anthracene, fluoranthene, pyrene, benzo[*a*]anthracene, chrysene, benzo[*b*]fluoranthene, benzo[*k*]fluoranthene, benzo[*a*]pyrene, indeno[1,2,3-*cd*]pyrene, dibenz[*a*,*h*]anthracene, benzo[*g*,*h*,*i*]perylene, benzo[*e*]pyrene, dibenz[*a*,*c*]anthracene, perylene, dibenz[*a*,*i*]pyrene, and coronene. Samples were collected over a 24-hr averaging period every 6 days starting on 31 March 1998 until 8 September 1998. PAHs in PM_10_ were measured using thin-layer chromatography and spectral luminescence detection according to standard Ukrainian methods at the Ukrainian Scientific Centre for Hygiene in Kiev (Poshyvanyk 1999).

### Statistical analysis.

All statistical analyses were performed using SPSS (SPSS Inc., Chicago, IL, USA) and PEPI (Programs for Epidemiology; USD Inc., Stone Mountain, GA, USA). We assessed distributions of 1-OHP for normality. Because 1-OHP exhibited non-normal distributions, exposure data were log-transformed to better approximate the assumed normality of the statistical tests. We calculated descriptive statistics, specifically the mean, median, SD, and geometric mean, for 1-OHP biomarker data and age of study participants. Means and SDs of stratified data controlling for resident city and second-hand smoke exposure status were also estimated. We assessed differences in means with Student’s *t*-test, using log-transformed data. The result was considered statistically significant if the *p*-value was equal to or less than 0.05.

## Results

### Study participants.

Urine samples for 1-OHP analysis were collected in 42 children from Kiev and 48 children from Mariupol. A description of the participants for both cities is given in Table 2. The percentage of males is slightly higher in the Mariupol group (50% vs. 44% in Kiev). The mean age of children from both cities is similar (3.0 vs. 3.1 years of age). A slightly higher percentage of children lived with smokers in Mariupol (46%) compared with Kiev children (37%).

### 1-OHP results for Mariupol and Kiev.

Descriptive statistics of 1-OHP of children living in each city are presented in Table 3. To adjust for individual differences in spot urine concentrations, creatinine-adjusted 1-OHP (1-OHP/creatinine) concentrations are shown along with the unadjusted values. Because not all samples were of sufficient quantity to test for both 1-OHP and creatinine, a subset of samples had data for both parameters and thus a smaller sample size for creatinine-adjusted 1-OHP results (Mariupol, *n* = 32; Kiev, *n* = 41). As shown in Table 3, the mean 1-OHP for Mariupol was significantly higher than the Kiev mean, for both adjusted (0.69 vs. 0.34 ng/mL; *p* < 0.0001) and unadjusted (0.52 vs. 0.30 ng/mL; *p* = 0.002) data sets. Tests of statistical significance were done on log-transformed data to meet assumptions of normality.

**Figure 3:**
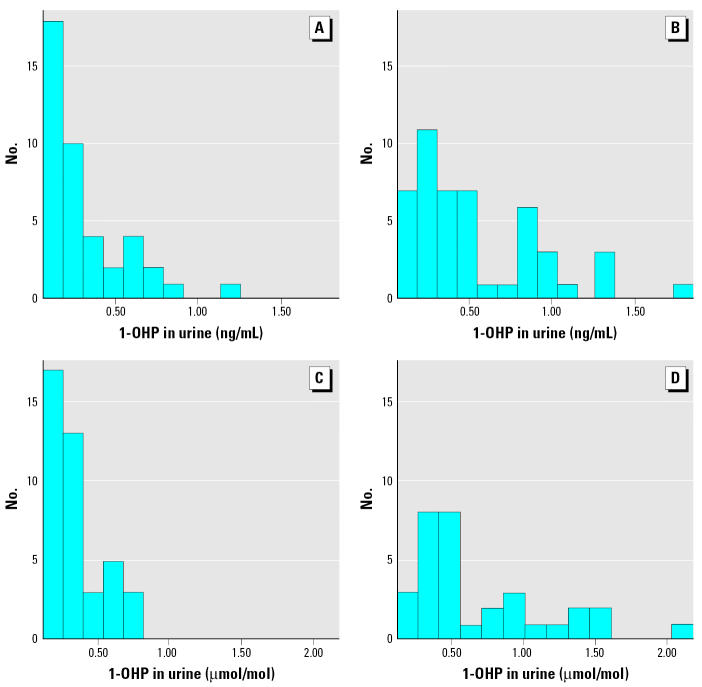
Histograms of creatinine-unadjusted and adjusted 1-OHP concentrations by city. (*A*) Kiev, ng 1-OHP/mL urine. (*B*) Mariupol, ng 1-OHP/mL urine. (*C*) Kiev, μmol 1-OHP/mol creatinine. (*D*) Mariupol, μmol 1-OHP/mol creatinine.

**Figure 4:**
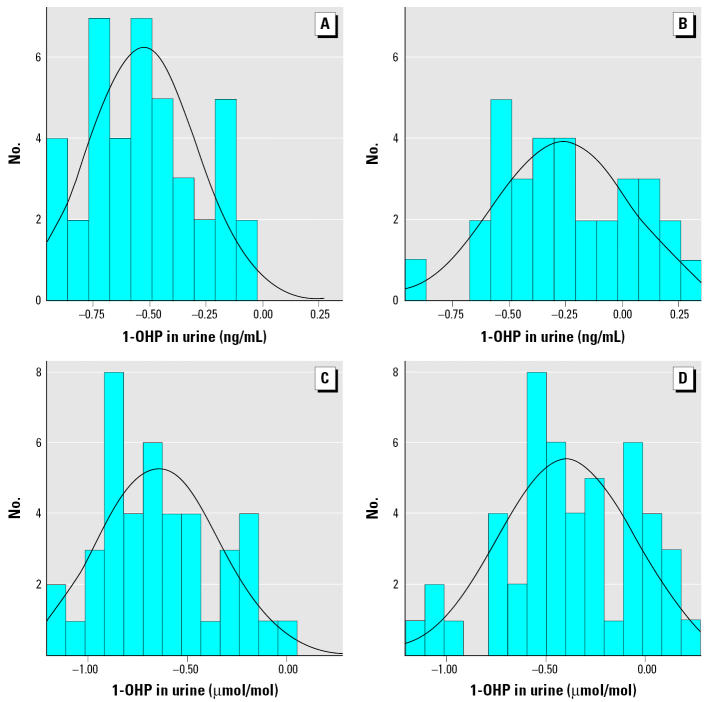
Log-transformed 1-OHP concentrations by city. (*A*) Kiev, creatinine-adjusted log-transformed 1-OHP ng/mL. (*B*) Mariupol, creatinine-adjusted log-transformed 1-OHP ng/mL. (*C*) Kiev, unadjusted log-transformed 1-OHP μmol/mol. (*D*) Mariupol, unadjusted log-transformed 1-OHP μmol/mol. Normal curve is superimposed.

1-OHP distributions, both as raw and as log-transformed data, are presented in Figures 3 and 4, respectively. These distributions illustrate significant differences in biologic exposure levels of children living in these two Ukrainian cities with different PAH sources. Log-transformed data visually present the validity of normality assumption for the statistical tests performed.

### Effect of sex.

When both sexes have been tested, previous studies have shown that 1-OHP tends to be higher in males than in females (Jongeneelen 1994; Siwinska et al. 1998, 1999). Table 4 presents 1-OHP concentrations by sex and city. No significant differences in 1-OHP mean concentrations by sex were found, for adjusted or unadjusted (and log-transformed) data. In addition, females had higher mean 1-OHP concentrations than did males, although the difference was not statistically significant.

### ETS exposure.

To examine the effect of second-hand smoke exposure on biologic exposure levels, we performed a stratified analysis of 1-OHP urinary levels and city of residence (Table 5). Even after stratifying on exposure to second-hand smoke, Mariupol children had mean 1-OHP levels more than twice as high as those of Kiev children using the log-transformed 1-OHP data (*p* = 0.004). Although there were no statistically significant differences in 1-OHP levels between children exposed and unexposed to second-hand smoke within either city, there was a statistically significant difference in 1-OHP concentrations if exposed to ETS, when using the combined city log-transformed data (*p* = 0.04). In addition, we used a regression model where association between the independent variables of second-hand smoke exposure and city of residence and the dependent variable of 1-OHP concentrations was assessed. Although passive smoking exposure was associated with 1-OHP, it was not statistically significant (*p* = 0.07), as shown in Table 6. The regression analysis also revealed that resident city was a highly significant variable (*p* < 0.001).

### PAHs in ambient air.

Twenty-two particulate phase PAH samples were collected approximately 2 weeks after biomarker data collection. The BaP range was 6.9–18.8 ng/m^3^, with a mean of 11.8 ng/m^3^. Pyrene concentrations ranged from 0.02 (half of the detection limit) to 20.6 ng/m^3^, with a mean of 7.6 ng/m^3^.

## Discussion

Mariupol children living within 3 miles of a steel mill and coke oven have the highest mean urinary concentrations of 1-OHP yet reported for young children. The upper end of the Mariupol 1-OHP distribution overlaps with reported values for occupationally exposed adults and smokers (Levin 1995). There was a statistically significant difference between children living in the point-source–affected area versus those living in the urban high-traffic environment of Kiev, the capital city of Ukraine. This is one of the first studies investigating children living in close proximity to steel mills and coke ovens, which are significant environmental sources of PAHs. Most other studies have focused primarily on children exposed to PAHs from traffic and/or dietary sources of PAHs (Fiala et al. 2001; Kanoh et al. 1993; Vyskocil et al. 2000; Zhao et al. 1990).

Several of the earlier studies in children have shown that diet is the most significant contributor to 1-OHP (Fiala et al. 2001; Vyskocil et al. 2000), with some acknowledging that any environmental component was too small to clearly assess its contribution (van Wijnen et al. 1996). We did not assess dietary contributions to 1-OHP or have sufficient environmental monitoring data to perform a multimedia exposure analysis. However, it seems probable that the increased PAH exposure from local industries, in general, contributes significantly to 1-OHP levels in these children, compared with those who live without environmental PAH exposure of this magnitude.

Only a few studies (Jongeneelen 1994; Siwinska et al. 1998) have investigated the effect of sex on children’s 1-OHP concentrations. Other studies either did not report any effects of sex or the association could not have been assessed because only one sex was tested (Chuang et al. 1999; Fiala et al. 2001; Kanoh et al. 1993; Karahalil et al. 1998; Northridge et al. 1999; van Wijnen et al. 1996; Vyskocil et al. 2000). In both of the cases where an effect of sex was seen (males had higher 1-OHP concentrations), the association was significant only when using unadjusted (for creatinine) and non-log-transformed data. Our findings of no effect of sex were based on log-transformed 1-OHP data, both adjusted and unadjusted. We observed higher 1-OHP levels in females, but the difference was not statistically significant, using creatinine-adjusted, unadjusted, or log-transformed data.

ETS or second-hand smoke contains PAHs and can thus be an important contributor to 1-OHP levels. Occupational studies have shown that smokers have a significantly higher amount of 1-OHP than do nonsmokers (Jongeneelen 2001). Some studies of children have tried to account for tobacco smoke exposure by measuring PAHs in indoor air or via an exposure questionnaire (Chuang et al. 1999; Fiala et al. 2001; Siwinska et al. 1998, 1999; van Wijnen et al. 1996). Second-hand smoke exposure was significant only when looking at the total group of children, comparing mean 1-OHP in those exposed with those unexposed, but not within each city. Our results did not clearly show an effect of ETS likely because the environmental industrial exposure was so predominant.

Because the air quality data were not concurrently collected with the biomarker data in Mariupol, because of logistical reasons, the utility of the PAH air quality data in interpreting biologic exposure information was limited. In addition to study analyses of ambient air, Hydromet (the Ukrainian state environmental control organization) routinely collected ambient air data in both Mariupol and Kiev [Ministry for Environmental Protection and Nuclear Safety of Ukraine (MEPNS) 1998]. Annual averages for the following contaminants in Mariupol for 1998 were sulfur dioxide, 0.20 mg/m^3^; nitrogen dioxide, 0.04 mg/m^3^; BaP, 3.8 ng/m^3^; and “dust,” 0.20 mg/m^3^. For Kiev, the 1998 annual averages were SO_2_, 0.013 mg/m^3^; NO_2_, 0.07 mg/m^3^; BaP, 1.8 ng/m^3^; and “dust,” 0.10 mg/m^3^. Previous analyses of environmental sources in Mariupol indicate that the two steel mills and coking facility combined are responsible for > 99% of air pollutants emitted from stationary sources in the city (MEPNS 1998). For comparison, we compared Ukrainian Hydromet data with U.S. EPA modeled estimates of toxic air pollutants using 1996 air data (MEPNS 1998; U.S. EPA 2003b). One of the contaminants estimated was “7-PAH,” which is composed of benz[*a*]anthracene, benzo[*b*]fluoranthene, benzo[*k*]fluoranthene, benzo[*a*]pyrene, chrysene, dibenz[*a*,*h*]anthracene, and indeno[1,2,3-*cd*]pyrene. The BaP concentration in Mariupol alone compares with the 95th percentile of seven PAHs from U.S. EPA modeled estimates (0.0038 μg/m^3^), indicating the high degree of PAH contamination in the air compared with the United States.

This study indicates that children living next to industrial point sources of PAHs can have very high 1-OHP levels. Mariupol children, who live near significant point sources of PAH contamination, had elevated 1-OHP concentrations compared with other measured Ukrainian children without such environmental contamination. Future research should focus further on this highly exposed population to better understand their sources of PAH exposure, including their diets. A more detailed and multimedia exposure analysis would identify those exposure pathways contributing most to total body burden and better define exposure reduction and risk management options. In addition, more specific and long-term biomarkers, such as PAH–DNA adducts, could be employed to link with measures of health effects.

## Figures and Tables

**Figure 1:**
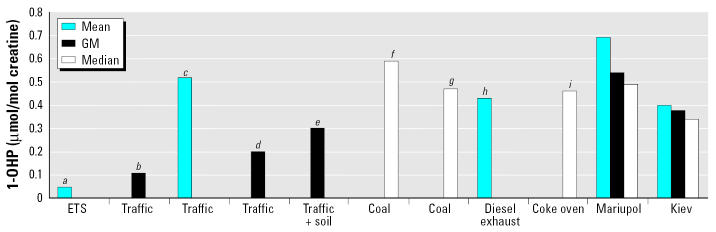
Literature summary of 1-OHP concentrations in children. **a**Chuang et al. 1999 ,**b**Fiala et al. 2001, **c**Zhao et al. 1990, **d**Vyskocil et al. 2000, **e**van Wijnen et al. 1996, **f**Siwinska et al. 1998, **g**Siwinska et al. 1999, **h**Northridge et al. 1999, **i**Jongeneelen et al. 1994.

**Table 1:** Review of reported children’s urinary 1-OHP concentrations.

Study	PAH source	Age	Study population	Urinary 1-OHP concentrations	Range
Three areas in Tokyo, Japan (Kanoh et al. 1993)	Urban/traffic	4th, 5th, and 6th graders	Two groups of 37–70 children “approximately equal numbers of boys and girls”	Highest mean reported in study: 21.1 ng/dL (Itabashi group; summer sample, July; 6th graders)	Mean, 9.8–21.1 ng/dL
Two areas in North Carolina, USA (Chuang et al. 1999)	Multiple (no identified industries)	2–4 years	24 total; male, 13; female, 11	0.049 μmol/mol creatinine (mean)	0.008–0.18 μmol/mol creatinine
				0.13 ng/mL (mean)	0.009–1.23 ng/mL
Two cities in the Czech Republic (Fiala et al. 2001)	Traffic	3–6 years	25 in “polluted” area 32 in “nonpolluted” area Sex not specified	“Polluted area” geometric mean (summer/evening sample highest): 0.108 μmol/mol creatinine	0.021–0.495 μmol/mol
				“Nonpolluted area” geometric mean (summer/morning sample highest): 0.078 μmol/mol creatinine	0.018–0.281 μmol/mol creatinine
Three areas of Silesia, Poland (Siwinska et al. 1999)	Heating, coal- burning stoves, and ETS	7–8 years	412 total	Medians (μmol/mol creatinine):	Not provided
			Bytom (urban industrialized) 78	Bytom, 0.47	
			Dabrowa Gornicza (industrialized) 72	Dabrowa Gornicza, 0.23	
			Pilica (rural commune) Sex not specified	Pilica, 0.38	
One area of Silesia, Poland (Siwinska et al. 1998)	ETS, indoor coal burning	8 years	30 total (6 days of sampling in the morning) Both sexes tested	Highest and lowest medians were 2.30–3.95 nmol/L	0.19–26.15 nmol/L
				0.28–0.59 μmol/mol creatinine	0.07–3.62 μmol/mol creatinine
Five areas in the Netherlands (van Wijnen et al. 1996)	Ambient air, mine tailings in soil	1–6 years	644 total Both sexes tested	0.34 μmol/mol creatinine (mean) 2.06 nmol/L (mean)	0.00–7.15 μmol/mol creatinine 0.05–47.26 nmol/L
Harlem, New York, USA (Northridge et al. 1999)	Urban sources, diesel exhaust	12–14 years	21 total Both sexes tested	Mean for the group: 0.43 pmol/mL	0.05–1.40 pmol/mL
Two cities in Turkey (Karahalil et al. 1998)	Occupational: engine repair shops	13–18 years	61 (exposed workers)	Mean for exposed: 4.71 ± 0.53 μmol/mol creatinine	For exposed: 0.80–23.90 μmol/mol creatinine
			30 (nonexposed workers)	Mean for nonexposed: 1.55 ± 0.28 μmol/mol creatinine	
Two areas in Montreal, Canada (Vyskocil et al. 2000)	Traffic	3–6 years	24 total children in kindergarten Sex not specified	Polluted area (morning; geometric mean): 0.20 μmol/mol creatinine	0.002–0.77 μmol/mol creatinine
				Nonpolluted area (evening; geometric mean): 0.13 μmol/mol creatinine	0.03–0.26 μmol/mol creatinine
Three cities in China (Zhao et al. 1990)	Urban sources	Primary school age	15 girls	0.52 μmol/mol creatinine (mean)	0–1.2 μmol/mol creatinine (estimated from figure)
Bytom, Upper Silesia, Poland (Jongeneelen 1994)	Coke ovens, indoor coal burning	8.5 years (mean)	148 total; male, 76; female, 72	0.46 μmol/mol creatinine (median) Male: 0.66 μmol/mol creatinine Female: 0.59 μmol/mol creatinine	0.09–6.99 μmol/mol creatinine

**Table 2:** Description of study population.

	Kiev (*n* = 43)	Mariupol (*n* = 48)
Age (years)		
Mean ± SD	3.1 ± 0.1	3.0 ± 0.2
Range	2.7–3.3	2.7–3.4
Sex		
Percent male	44	50
Percent female	56	50
Smoker in the home (%)	37	46

**Table 3:** 1-OHP concentrations in Ukrainian children.

1-OHP	Kiev	Mariupol
Unadjusted (ng/mL)		
No.	42	48^a^
Range	0.06–1.17	0.07–1.85
Mean	0.30	0.52^b^
Median	0.22	0.39
SD	0.24	0.39
Adjusted (μmol/mol creatinine)		
No.	41	32^a^
Range	0.11–0.81	0.12–2.18
Mean	0.34	0.69^c^
Median	0.28	0.49
SD	0.20	0.50

^a^The difference in sample sizes between creatinine-adjusted and unadjusted 1-OHP is due to the incomplete number of creatinine results obtained for all children.

^b^*p* = 0.002; using log-transformed data, *p* = 0.001.

^c^*p* < 0.001; using log-transformed data, *p* < 0.0001.

**Table 4:** 1-OHP concentrations by sex and city (μmol/mol creatinine, mean ± SD).

	Kiev	Mariupol
Male	0.31 ± 0.21	0.62 ± 0.45
Female	0.37 ± 0.19	0.74 ± 0.53

No significant differences were seen with sex, either using data above or with log-transformed data (not shown).

**Table 5:** 1-OHP concentrations by city and ETS exposure.

City	Smokers present in the home	Mean 1-OHP (μmol/mol creatinine)	No.	SD
Kiev	Yes	0.37	14	0.16
	No	0.33	26	0.21
	Total	0.34	40	0.20
Mariupol	Yes	0.83^a^	15	0.59
	No	0.56	17	0.37
	Total	0.69	32	0.50
Total (both cities)	Yes	0.61^b^	29	0.50
	No	0.42	43	0.30
	Total	0.50	72	0.40

^a^*p* = 0.004, comparing exposed children in Mariupol versus exposed children in Kiev.

^b^*p* = 0.04, comparing all children exposed to ETS with those unexposed (test done using log-transformed 1-OHP data). No significant differences were seen with ETS exposure within each city.

**Table 6:** Regression analysis: effect of secondhand smoke exposure and resident city on 1-OHP.

	B	SE	Significance
(Constant)	−0.57	0.05	
City of residence	0.25	0.06	0.000*
Smoker(s) present in the home	0.12	0.07	0.073

Mariupol as city of residence is the exposed group.

*p < 0.001.
